# Electricity-assisted production of caproic acid from grass

**DOI:** 10.1186/s13068-017-0863-4

**Published:** 2017-07-11

**Authors:** Way Cern Khor, Stephen Andersen, Han Vervaeren, Korneel Rabaey

**Affiliations:** Department of Biochemical and Microbial Technology, Centre for Microbial Ecology and Technology (CMET), Ghent University, Coupure Links 653, 9000 Ghent, Belgium

**Keywords:** Grass, Lactic acid, Caproic acid, Decane, Fermentation, Chain elongation, Electrolysis

## Abstract

**Background:**

Medium chain carboxylic acids, such as caproic acid, are conventionally produced from food materials. Caproic acid can be produced through fermentation by the reverse β-oxidation of lactic acid, generated from low value lignocellulosic biomass. In situ extraction of caproic acid can be achieved by membrane electrolysis coupled to the fermentation process, allowing recovery by phase separation.

**Results:**

Grass was fermented to lactic acid in a leach-bed-type reactor, which was then further converted to caproic acid in a secondary fermenter. The lactic acid concentration was 9.36 ± 0.95 g L^−1^ over a 33-day semi-continuous operation, and converted to caproic acid at pH 5.5–6.2, with a concentration of 4.09 ± 0.54 g L^−1^ during stable production. The caproic acid product stream was extracted in its anionic form, concentrated and converted to caproic acid by membrane electrolysis, resulting in a >70 wt% purity solution. In a parallel test exploring the upper limits of production rate through cell retention, we achieved the highest reported caproic acid production rate to date from a lignocellulosic biomass (grass, via a coupled process), at 0.99 ± 0.02 g L^−1^ h^−1^. The fermenting microbiome (mainly consisting of *Clostridium* IV and *Lactobacillus*) was capable of producing a maximum caproic acid concentration of 10.92 ± 0.62 g L^−1^ at pH 5.5, at the border of maximum solubility of protonated caproic acid.

**Conclusions:**

Grass can be utilized as a substrate to produce caproic acid. The biological intermediary steps were enhanced by separating the steps to focus on the lactic acid intermediary. Notably, the pipeline was almost completely powered through electrical inputs, and thus could potentially be driven from sustainable energy without need for chemical input.

**Graphical abstract:**
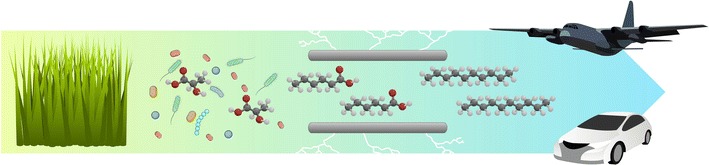
Microbial and electrochemical production of lactic acid, caproic acid and decane from grass.

**Electronic supplementary material:**

The online version of this article (doi:10.1186/s13068-017-0863-4) contains supplementary material, which is available to authorized users.

## Background

Caproic acid is a medium-chain carboxylic acid, its demand has been growing due to its application as chemical commodity, feed additive and more recently as bio-based fuel precursor. The medium-chain carboxylic acid market (caproic acid, caprylic acid, capric acid, and lauric acid) is predicted to reach USD 1.25 billion globally by 2020 [1]. Generally, these medium chain fatty acids (MCFA) are derived from triglycerides of coconut and palm oil by fractional distillation, ozonolysis or catalytic reduction processes [2]. MCFA can also be microbially synthesized from alcohols and carboxylic acids through fermentation. Zhu et al. [3] recently demonstrated that a solution of lactic acid could enable caproic acid production through the microbial reverse β-oxidation pathway.

Lactic acid is typically formed during the fermentation/ensiling of grass. Grass is a widely available substrate presently underused and often overlooked as a carbon source. In the US, grasslands represent an estimated 2.51 × 10^6^ km^2^ of available biomass [4], which is either ensiled or lowly used. A grass-based fermentation can lead to the formation of lactic and acetic acid. In an uncontrolled pH, mesophilic fermentation of grass, a concentration of 12.6 g L^−1^ lactic acid can be reached, usually along with 2.0 g L^−1^ of acetic acid [5]. Lactic acid and acetic acid are highly soluble in water, which makes the downstream extraction of lactic acid energy intensive. More hydrophobic products such as caproic acid can be produced through the reverse β-oxidation pathway with ethanol as the most common reducing substrate [6] and acetic acid as the electron acceptor. Studies have been carried out with pure cultures such as *Clostridium kluyveri* using ethanol [7, 8] and *Megasphaera elsdenii* using sugars and lactic acid [9]. Caproic acid production from lignocellulosic material [10] and a route using lactic acid was first considered in 1956 [11], with attention returning to this process in recent years, through the reverse β-oxidation pathway pure culture fermentations [12], and mixed culture community fermentations [3, 13–17]. Zhu et al. proposed the stoichiometry of caproic acid formation via lactic acid as 3 CH_3_CHOHCOO^−^ + 2 H^+^ → CH_3_(CH_2_)_4_COO^−^ + H_2_O + 2 H_2_ + 3 CO_2_, with Gibbs free energy of −123.1 kJ mol^−1^ [3].

The hydrophobicity and low water solubility of caproic acid allow extraction of caproic acid from the fermentation broth by phase separation. If the concentration of caproic acid in its protonated form exceeds the solubility limit (11.0 g L^−1^ at 20 °C), caproic acid will form an immiscible layer and phase separate from the fermentation broth along with other hydrophobic chemical species. Recently, Xu et al. [18] managed to extract n-caproic acid from a bioreactor broth using an in-line membrane electrolysis system, while minimizing caustic and acidic dosing with electrochemistry.

**Fig. 1:**
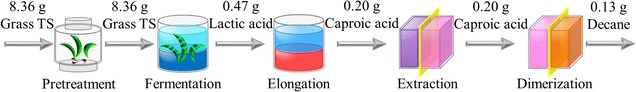
Overall process of conversion of grass into lactic acid, caproic acid and decane

Here, we studied the feasibility of a complete pipeline for caproic acid generation from grass via microbial fermentations and electricity-driven processes alleviating the use of chemicals, and completely avoiding energy intensive dewatering and distillation steps on the carboxylic acid intermediates. Lactic acid fermentation broth from grass elongates to caproic acid by reverse β-oxidation. The caproic acid extracts via membrane electrolysis delivering the acid concentration of caproic acid. As final proof of concept, we also converted caproic acid to decane via Kolbe electrolysis. For each process rate, efficiencies and intermediary concentrations were determined. The overall process is presented in Fig. 1.

## Results and discussion

### Semi-continuous fermentation of lactic acid from grass

**Fig. 2:**
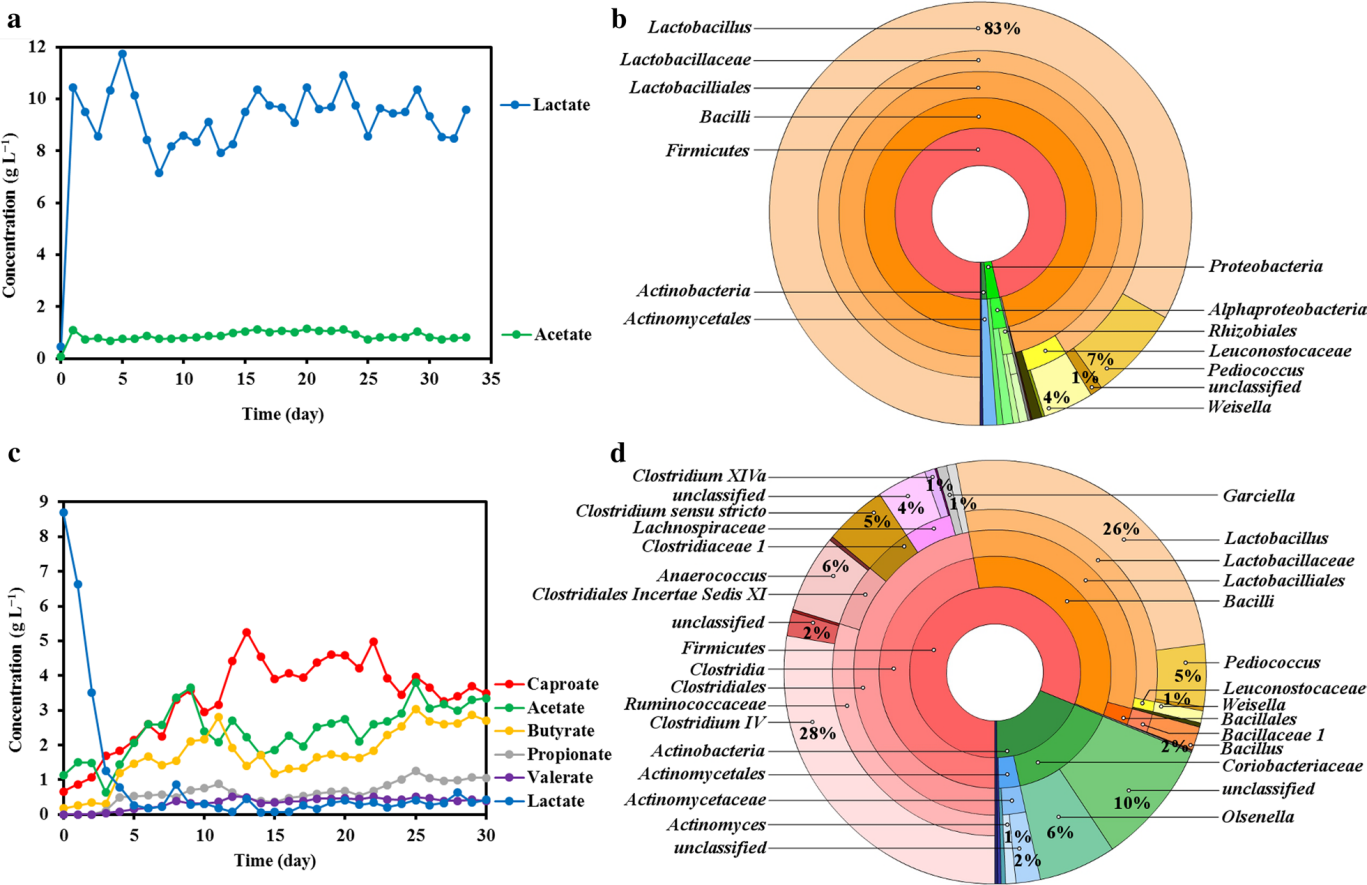
Carboxylate profile and bacterial community of fermentation and elongation system: **a** carboxylate profile of fermentation system, **b** bacterial community for fermentation system, **c** carboxylate profile of elongation system, **d** bacterial community for elongation system. The concentric circles represent the bacterial taxonomy from phylum (*centre*) to genus (*outermost*). *Each band* indicates the proportion of the bacteria. *One colour* is dedicated to each classification

Semi-continuous fermentation of lactic acid using grass as substrate was performed for 33 days, with a solids and liquid retention time of 2 days. The lactic acid concentration stabilized at 9.36 ± 0.95 g L^−1^, and the acetic acid concentration at 0.90 ± 0.14 g L^−1^ (Fig. 2a). The pH of the fermentation broth was between 4.5 and 5. The lactic acid concentration rapidly reached its final concentration within the first day of fermentation, indicating that the native microorganisms of grass were active as there was no lag time for lactic acid production. In this study, lactic acid and acetic acid were the main products during fermentation due to low pH and short retention time.

The conversion of organic compounds to lactic acid was low relative to our previous study [5] (0.065 g g^−1^ in this study compared to 0.136 g g^−1^ grass volatile solid), while the rate of lactic acid production was 0.197 g L^−1^ h^−1^ in this study. The conversion rate and efficiency of biomass to lactic acid were relatively low in this study, which is likely due to the limited pretreatment and reactor design involving a higher ratio of solution volume relative to mass of grass. Pretreatment (e.g. grinding, steam-explosion, alkaline) can be performed to improve the biomass biodegradability, while taking into consideration the cost-benefit-sustainability nexus. For instance, mild pretreatment methods such as lime pretreatment can be applied without introducing excessive cost, to improve conversion efficiency and production rate, and minimize carbon loss [19]. In earlier studies, it was shown that pretreatment, such as extrusion, can improve the degradability of the grass [19]. At the time of this manuscript, the equipment was not available; however, similar results to Khor et al. [5] can be expected, where a conversion increase of 109% was noted. While pretreatment can enhance biomass biodegradability, it is also necessary to consider the biological impact of the pretreatment. Phenolic compounds and furfural can be released from lignin if the pretreatment is severe (e.g. high temperature, low pH), which can then interfere with fermentative processes and inhibit the growth of microorganisms. Hence, a substrate purification step (e.g. metal catalysts, adsorption or chemical oxidation) to remove inhibitory compounds prior to biological processes could be beneficial to improve product yield. In our study, the protein fraction of grass is assumed to be well retained and not degraded after fermentation due to the short retention time and low pH of the process, and no ammonia gas was detected. In a 2nd generation biorefinery process, complete conversion of lignocellulosic biomass into a single targeted compound is highly unlikely due to the complexity of the substrate. Instead, available fractions of the biomass can be converted into desired compounds at high rate, with the remainder recovered as by-products. For instance, in this case, one could foresee that the hemicellulose/cellulose fraction can be converted into lactic acid, while the remaining protein/amino acid fraction is recovered via an alternate route, such as the Grassa process [20].

For lactic acid fermentation, community analysis indicated that over time, the population became enriched with lactic acid bacteria such as *Lactobacillus* spp. (83% relative abundance), *Pediococcus* and *Weissella* spp. (Fig. 2b). *Lactobacillus*, *Pediococcus* and *Weissella* species are known for their ability to utilize polysaccharides and monosaccharides with lactic acid as a key outcome, as was observed in our experiments [5, 21].

### Semi-continuous elongation of caproic acid through lactic acid

When the grass fermentation effluent was fed into the elongation system, lactic acid was consumed, producing caproic acid (C6), as shown in Fig. 2c, along with butyric acid (C4) and acetic acid (C2). Enanthic acid (C7) and caprylic acid (C8) were not detected, unlike other chain elongation systems [16]. The caproic acid concentration was 4.09 ± 0.54 g L^−1^ (9.03 ± 1.19 g COD L^−1^), and 49 ± 9% of products formed was caproic acid during the stable phase (30 day operation, Fig. 2c). The pH of the elongation broth was between 5.5 and 6.2, showing the pH increasing effect of chain elongation.

The stoichiometry of 3 mol lactic acid forming 1 mol caproic acid as proposed by Zhu et al. [3] was employed to assess the caproic acid production in this study. In terms of lactic acid and caproic acid balance, on average 4.68 ± 0.47 g L^−1^ (53 ± 5 mM) of lactic acid was fed and the average caproic acid production was 2.05 ± 0.27 g L^−1^ (18 ± 2 mM). This fits well with the stoichiometry of 3 mol lactate forming 1 mol caproate, or a consumption of 2.32 g lactate per g caproate formed. Lactic acid generated from a low-value substrate can thus act as an effective intermediary for caproic acid production. While caproic acid production can also be achieved via ethanol, this tends to bind one to an integrated biorefinery or other ethanol producing (bio-)processes. Under different circumstances (e.g. substrate availability, market considerations), one can foresee that both pathways (i.e. chain elongation using lactate or ethanol) are interesting.

In the elongation system, a different community developed from the initial fermentation, despite influx via the substrate. *Firmicutes* dominated the population (81% relative abundance), in which *Clostridium* IV- and *Lactobacillus*-related species were most prevalent. *Coriobacteriaceae* and *Anaerococcus* spp. were other highly abundant species (Fig. 2d). *Clostridium* species, including *Clostridium* IV, *Clostridium Sensu Stricto* and *Clostridium* XIVa spp. identified in this study, are the archetype organisms known to perform reverse β-oxidation [22, 23], and also utilize lactic acid. Although being weakly fermentative, *Anaerococcus* spp. is known to be able to metabolize carbohydrate and produce butyric acid and lactic acid as major metabolic end products. Some genus have butyric acid and caproic acid as major end products [23], which fits very well in this context. *Coriobacteriaceae* spp. has been mostly reported to be saccharolytic; however, its genus such as *Olsenella* spp. is able to convert glucose into lactic acid as major product [24]. The family of *Actinomycetaceae* spp. are also able to produce lactic acid and acetic acid under fermentative condition [25]. The bacterial composition of the inoculum and the elongation system were similar (Additional file 1: Fig. S1a; Fig. 2d). This showed that introduction of bacterial community from the fermentation system did not change the bacterial community of the elongation system significantly.

### Maximum rate of caproic acid production and maximum concentration of caproic acid test

**Fig. 3:**
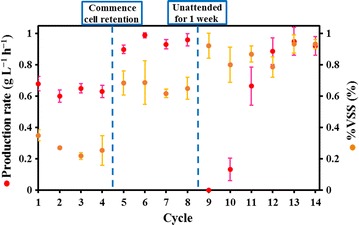
Production rate of caproic acid (g L^−1^ h^−1^, *red circles*) and percentage of volatile suspended solids (%VSS, *orange circles*) of elongation broth during maximum rate of caproic acid production test (*error bars* represent the standard deviation of triplicates)

The highest rate of caproic acid production reached was 0.99 ± 0.02 g L^−1^ h^−1^, when the cells were retained (Fig. 3—cycle 6). The system was left unattended for 1 week to test the resilience of the elongation microorganisms, and the production rate recovered to the maximum rate after 4 cycles of operation (Fig. 3—cycle 9 to 12). Table 1 compares different feed sources and maximum rates of medium chain fatty acids produced for microbial chain elongation process through a select screening of literature.

**Table 1:** Microbial chain elongation operation and maximum rate of medium chain fatty acid production

Feed source	Operation	Culture	pH	Maximum rate of production (g L^−1^ h^−1^)	Reference
Synthetic medium containing acetate, ethanol, and yeast extract, with CO_2_ gas flow	Packed bioreactor	Mixed (non-sterilized, mainly *Clostridium kluyveri*)	6.5–7.2	2.39 (medium chain fatty acids)	[26]
Grass fermentation effluent containing lactic acid	Semi-continuous	Mixed (non-sterilized, mainly *Clostridium cluster IV*)	5.5–6.2	0.99 (caproic acid)	This study
Synthetic medium containing galactitol, yeast extract, with in situ extraction	Batch	Pure (*Clostridium* sp. BS-1)	6.5	0.34 (caproic acid)	[12]
Dilute ethanol and acetate, with in-line extraction	Continuous	Mixed (non-sterilized, mainly *Clostridium* spp.)	5.2–5.5	0.33 (caprylic acid)	[27]
Synthetic medium containing acetate and ethanol	Semi-continuous	Mixed (non-sterilized, mainly *Clostridium kluyveri*)	7	0.20 (caproic acid)	[28]
Castor oil	Continuous	Mixed (non-sterilized, mainly *Clostridium kluyveri*)	6.5–7	0.19 (caproic acid)	[29]
Yeast fermentation beer	Continuous	Mixed (non-sterilized, mainly *Clostridium kluyveri*)	5.5	0.14 (caproic acid)	[30]
Diluted yellow water	Semi-continuous	Mixed (non-sterilized, mainly *Clostridium cluster IV*)	5.5–6.5	0.12 (caproic acid)	[3]

The maximum caproic acid production rate achieved in this study is high compared to literature, such as the study of Grootscholten et al. where 4.5 g L^−1^ day^−1^ (0.19 g L^−1^ h^−1^) was achieved with mixed culture up-flow anaerobic filter system using castor oil, at pH between 6.5 and 7 [29]. Comparable rates as reported here were thus far only obtained with synthetic media, with the highest from acetic acid and ethanol, 57.4 g L^−1^ day^−1^ (2.39 g L^−1^ h^−1^) [26], albeit with a considerable higher loading rate. Our value considers only the rate of the lactic acid-rich reactor effluent, and biomass hydrolysis is broadly recognized as a rate limiting step in conversion of ‘real’, complex substrates. Further, it is important to note that to achieve this rate, Grootscholten et al. used a neutral pH between 6.5 and 7.2 [26], thus requiring dosing of base which was not the case in our study.

Cell density is often unreported in mixed culture fermentations, and cell retention and maximizing the density in the fermenters can potentially improve conversion dramatically. The production rate achieved here is partially a result of high microbial density, lack of pH controlling salts such as sodium hydroxide and the used substrate (real versus synthetic). *Anaerococcus* spp. (32% relative abundance) became the most dominant species (Additional file 1: Figure S1b), during the maximum rate of caproic acid production test with cell retention, followed by *Lactobacillus* spp., while *Clostridium* IV spp. was merely 6% relative abundance. *Anaerococcus* are typically found in anaerobic conditions and were recently identified under methanogenic conditions [31], and have not been implicated in chain elongation to our knowledge. *Clostridium* IV spp. have been broadly implicated in reverse β-oxidation with lactic acid as an intermediary [3, 6, 17].

In order to determine the maximum concentration of caproic acid the community could sustain without a substrate limitation, an excess of lactic acid was fed to the fermenter. The maximum concentration of caproic acid reached was 10.92 ± 0.62 g L^−1^ (n = 3). By molar concentration, caproic acid consisted of 57 ± 4% of the products formed (Additional file 1: Figure S2), compared to 34 ± 9% during normal elongation operation in this study. While chain elongation is often preferred at neutral pH (6.5–7.2) to avoid toxicity of caproic acid, the highest concentration achieved in this study was under acidic conditions (pH 5.5–6), suggesting some tolerance of the community to the usually anti-microbial caproic acid. During the maximum concentration of caproic acid test in which excess lactic acid was provided, *Clostridium* IV spp. remained the most dominant (Additional file 1: Figure S1c), and other species reduced in terms of abundance. This provides additional evidence that *Clostridium* IV spp. are generating caproic acid through the lactic acid intermediate, and indicates that *Clostridium* IV spp. can tolerate a caproic acid concentration at least up to 10.92 g L^−1^, at the border of the solubility limit. The mixed population showed functional stability between experiments, while the community members differed.

### Electrochemical extraction of caproic acid and decane production through Kolbe electrolysis

Effluent from the elongation system was sent to the cathodic chamber of an electrochemical system for extraction and concentration of caproic acid, with an applied current of 0.4 A (resulting in 25 A m^−2^). When current was applied, the anion exchange membrane allowed anions such as carboxylates, sulphate, chloride and phosphate to migrate from the cathodic to anodic compartment through the membrane to complete the circuit. The pH dropped at the anodic compartment as protons were generated due to water electrolysis, and carboxylates formed their undissociated counterparts and thus accumulated in the anodic compartment. Over time, the caproic acid phase-separated from the anodic broth as the concentration exceeded its maximum (11 g L^−1^ at 20 °C in water), forming a lower density, hydrophobic layer on top of the solution (Additional file 1: Figure S3), similar to that of Xu et al. [18].

**Fig. 4:**
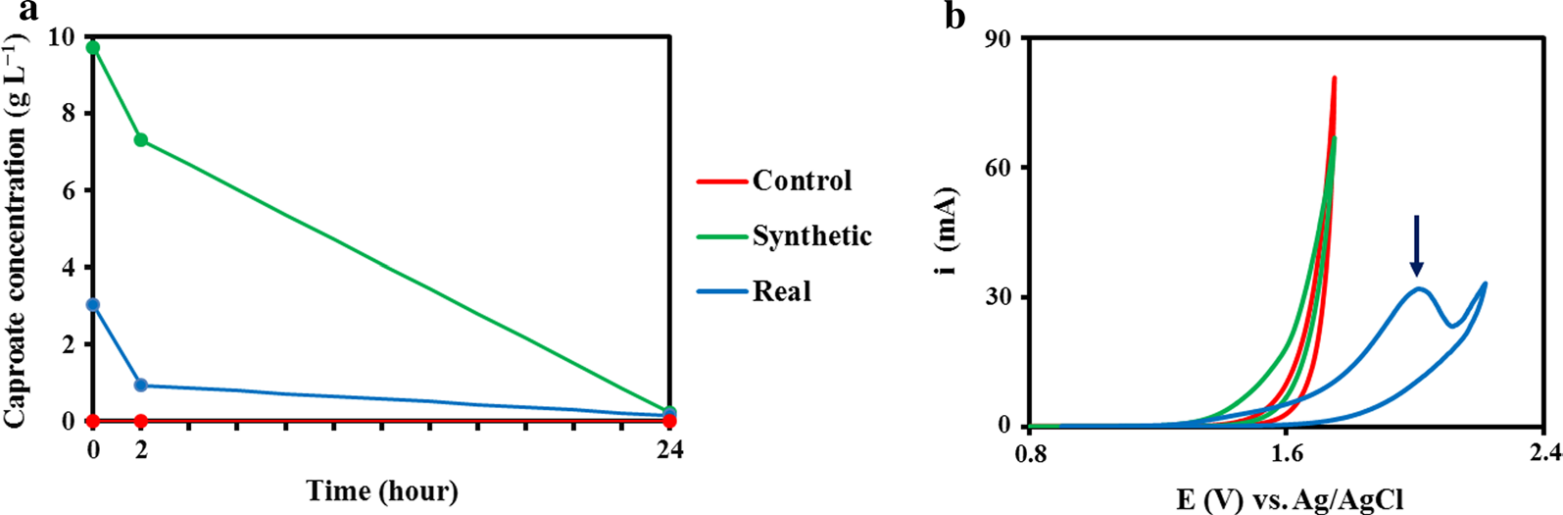
Caproate concentration and cyclic voltammetry of Kolbe electrolysis process: **a** Caproate concentration over the period of Kolbe electrolysis, and **b** cyclic voltammetry of solution before Kolbe electrolysis. Control (*red line*) contained only 0.5 M Na_2_SO_4_ in water, synthetic solution (*green line*) contains 0.1 M caproic acid and 0.5 M Na_2_SO_4_ in water, and real solution (*blue line*) was the anolyte from electrochemical extraction system

In parallel with the caproic acid extraction, the caproic acid concentrate was also tested for conversion to decane. Energy-dense alkanes are classically obtained from fossil fuels, but can also be generated electrochemically from carboxylic acids. Kolbe electrolysis was performed on the extracted product, introduced in the anolyte again to a concentration of 3.03 g L^−1^ (26 mM), and compared against a synthetic caproic acid electrolysis. The synthetic solution cyclic voltammetry (CV) was shifted earlier compared to the control, which suggested an anodic reaction apart than water electrolysis, while the real solution CV gave a distinctive peak for an unidentified peak for an anodic reaction (Fig. 4b). Increasing the current density beyond the mass transport limit can give rise to oxygen evolution rather than the desired Kolbe process, resulting in loss of efficiency. The electrolysis reaction resulted in decarboxylation and dimerization of caproic acid, leading to the production of decane in the organic phase. Caproic acid was electrolyzed at a rate of 1.21 g L^−1^ h^−1^ (0.202 kg m^−2^ h^−1^) for the control synthetic solution and 1.05 g L^−1^ h^−1^ (0.174 kg m^−2^ h^−1^) for the real product effluent solution, only 13% less than the control (Fig. 4a). Compared to literature, conversion rates of valeric acid were 1.9 kg m^−2^ h^−1^ assuming 100% Coulombic efficiency [32]. The coulombic efficiency was relatively low in this study, approximately ten times less, as the un-optimized process also contained other carboxylic acids and salts extracted alongside the target product. Furthermore, lower solubility of caproic acid compared to valeric acid also contributed to less reactants in the solution, which can decrease product yield [33–35]. The aqueous phase contains more impurities such as Cl^−^, ${\text{SO}}_{4}^{{2-}}$, acetic acid, butyric acid and others, which subsequently led to formation of other impurities and non-Kolbe products which warrants further investigation. NMR analysis reveals that 10% of the product was decane, and indicated that decane was not the sole product. A quantitative analysis was not possible due to small amount that could be sampled throughout the laboratory pipeline. In theory, a range of other Kolbe and non-Kolbe products such as 1-pentene, 2-pentanone, pentan-1-ol, and valeric acid can be formed; however, they could not be accurately identified in this study due to the limited sample amount and complexity of the mixture.

Compared to the literature, Kolbe electrolysis performed at current density of 0.13 A cm^−2^ and temperature of 293 K, using synthetic caproic acid solution and sono-emulsion system in the presence of 190 W cm^−2^ ultrasound, yields 24 ± 3% of the Kolbe dimer [36]. In this study, the efficiencies of conversion were lower, likely due to the complexity of the fermentation broth and the unoptimized conditions. Increasing current density can potentially result in a higher yield; Wadhawan et al. reported an improvement from 24 to 45% when current density was increased from 0.13 to 0.18 A m^−2^ [36]. Integration of power ultrasound can improve mass transport and help remove the products formed from the electrode surface, resulting in higher conversion efficiency. This study demonstrates that the mixed solution of caproic acid (alongside other carboxylic acids and other salts) can still be converted to alkanes, which is a promising route for carboxylic acid fermentation streams. The tendency for microbial communities to form side products is a challenging issue in biorefinery production from wastes, and effective conversion processes that are unimpeded by side products are valuable to explore.

### Grass to chemicals and fuels: preliminary economic assessment

Grass was used as substrate to produce chemicals such as lactic acid, caproic acid and decane. Here, the process was performed in discrete steps, whereas in future phases the caproic acid fermentation and extraction will be directly coupled, and potentially the Kolbe electrolysis can be performed directly in the anode of the extraction cell, pending reactor and electrode design. The system could thus be simplified to two fermenters and a coupled electrochemical system which will likely entail efficiency increases.

An economic estimate was made by considering the operating costs (Table 2). Pretreatment such as extrusion requires around 0.20 kWh kg^−1^ TS of grass [19], and extraction using membrane electrolysis needs approximately 2 kWh kg^−1^ carboxylic acid extracted. Kolbe electrolysis of valeric acid into octane requires around 1.64 kWh kg^−1^ octane, assuming 100% selectivity [32]. Here, 30% conversion of grass TS to lactic acid, and 50% conversion of caproic acid to decane are assumed, and an electricity price of 0.125 USD kWh^−1^. Production of 1 kg of lactic acid thus costs 0.33 USD. When further converted into caproic acid, the production cost increased to 0.44 USD and conversion to 1 kg decane incurred a cost of 1.29 USD kg^−1^ decane produced. Compared to the market price of the products, lactic acid (1.00 USD kg^−1^), caproic acid (1.60 USD kg^−1^) and decane (0.41 USD kg^−1^, as aviation fuel), one can see that caproic acid has a higher margin for profit; however, one must consider that these prices represent market ready chemicals with a relatively small market. Lactic acid can also be a lucrative product, although energy-intense product purification may be needed, generally in the form of distillation. Decane may seem at first glance to be a less attractive end product due to its low economic value at this point of time; however, the liquid fuel market is extensive and well entrenched, and its low-density and low-solubility make it a rather simple product to target in terms of process engineering. If caproic acid can be produced biologically in mass, one can foresee that decane production may become a more attractive end-point due to the much higher demand. Moreover, depending on the final use, the presence of trace compounds in the product can require extensive purification trains. Both energy and unit investments for removing trace impurities, for example removing short chain carboxylic acids including formic and acetic acids, damage the economic argument in producing specialty chemicals like lactic acid from sustainable resources, while this is less of an issue for liquid fuels. In this study, the cost assessment was simplified to give a rough estimate on the economics of each target product. The chemical and electricity prices were also assumed based on literature values and available data, which can differ significantly over time, location and market. Biomass conversion generally varies over a range of 10 to 60% depending on the substrate and efficiency of pretreatment [5, 37]. A consistent supply of biomass is also crucial and biomass storage is often foreseen for continuous biorefinery processes. For a complete and elaborate assessment, other elements including capital and labour costs, and transportation cost for both grass and product need to be taken into account. As in first generation ethanol biorefineries, the cost and sustainability of collection and transport can make or break the argument for the grass to chemicals and fuels process.

**Table 2:** Material, energy and costs required to produce 1 kg of lactic acid, caproic acid or decane

	Lactic acid	Caproic acid	Decane
Material (kg TS)			
Grass	*3.33*	*7.69*	*25.00*
Energy (kWh)			
Pretreatment	0.67	1.54	5.00
Extraction	2.00	2.00	2.00
Kolbe electrolysis	–	–	3.28
Total	*2.67*	*3.54*	*10.28*
Cost ($)			
Pretreatment	0.08	0.19	0.63
Extraction	0.25	0.25	0.25
Kolbe electrolysis	–	–	0.41
Total	*0.33*	*0.44*	*1.29*
Selling price ($ kg^−1^)	*1.00*	*1.60*	*0.41*

In 2005, it was estimated that 2.5 × 10^11^ kg of municipal solid waste was generated in the United States, in which a significant fraction of the organic material was yard trimmings [38]. Assuming 10% municipal solid waste is made up of yard trimming, this gives an estimation of 1 × 10^9^ kg (1.35 × 10^9^ L) of fuel which can optimistically be produced in a green manner, and contribute to the approximately 78 × 10^9^ L of aviation fuel consumption in year 2012 in the US [39]. Today, the market size and need for energy-dense liquid fuel combined with present non-sustainable fuel production practices will continue to drive development of sustainable fuel production processes outcome.

## Conclusions

Here we demonstrated that a lignocellulosic biomass such as grass can be converted without addition of chemicals with electrochemical processes into an energy-dense chemical, through the production and utilization of a lactic acid intermediary towards caproic acid. The system can produce up to a maximum concentration in the fermentation of 10.92 ± 0.62 g L^−1^ caproic acid and achieved a production rate of 0.99 ± 0.02 g L^−1^ h^−1^, unmatched thus far for real substrates and at pH values relevant for fermentation. Despite a lower amount of end product as compared to lactic acid, caproic acid is simpler to harvest through phase separation, avoiding heat-based extraction. This process also opens an avenue towards the sustainable production of decane, a Kolbe electrolysis derivative of caproic acid.

## Methods

### Substrate and microorganisms

Farmland grass was harvested from a meadow on January 2016 (East Flanders, Belgium). The grass was air-dried at 28 °C to total solid content of 0.836 ± 0.006 g g^−1^ grass (volatile solid 0.728 ± 0.011 g g^−1^ grass) and stored at 4 °C until used. To make the grass more accessible for fermentation, size reduction was performed with a blender (Philips Daily HR2100/90) before fermentation. The grass particles sizes were between 24 and 453 µm, with a mean of 98 ± 74 µm over 200 measurements. The bacterial culture for lactic acid fermentation was native to the grass itself without extra inoculation. The microbiome for elongation, dominated by *Clostridium*- and *Lactobacillus* spp.-related species, was obtained from a continuous reactor producing caproic acid from thin stillage containing both ethanol and lactate [40]. 25 mL of thin stillage reactor effluent was collected and centrifuged at 5000*g* for 5 min. The supernatant was removed and the pellet obtained was washed 3 times with tap water and dissolved in 10 mL tap water before it was used as inoculum for elongation test.

### Fermentation

The semi-continuous fermentation test for grass to lactic acid consisted of a 100-mL reactor filled with 50 mL of tap water and 10 g (wet weight) of grass. The grass was packed in 2 bags made of AISI 316 stainless steel mesh (44 µm mesh size, 33 µm wire thickness), each containing 5 g (wet weight) of grass. The experiment was run under anaerobic condition for 33 days at 32 °C. Steady-state values are reported as the average of 20 samples taken over a stable period (7% variation) of 20 days (i.e. 10 HRTs). The substrate in one of the stainless steel bags was replaced every day (2 days of solid retention time). Half of the liquid phase was removed and refilled with tap water (2 days of hydraulic retention time). The system was sparged with nitrogen each time during substrate and liquid replacement and flushed with nitrogen after substrate replacement to ensure anaerobic condition. Samples were collected every day for carboxylic acids analysis. Samples were collected at day 0 and 33 for bacterial community analysis. pH was between 4.8 and 5.8, and it was not adjusted or controlled throughout the experiment.

### Microbial elongation

#### Semi-continuous operation

The semi-continuous elongation test consisted of a 100-mL reactor filled with 50 mL of elongation broth. Half of the solution was replaced with effluent from the fermentation test every day, resulting in 2 days of hydraulic retention time (HRT). The experiment was run under anaerobic condition for 30 days at 32 °C. Steady-state values are reported as the average of 20 samples taken over a stable period (13% variation) of 20 days (i.e. 10 HRTs). Samples were collected every day for carboxylic acids analysis. The system was sparged with nitrogen each time during substrate replacement and flushed with nitrogen after substrate replacement. Samples were collected at day 0, 9, 16 and 30 for bacterial community analysis. pH was between 5.5 and 6.3, and was not adjusted or controlled throughout the experiment. At the end of experiment, the elongation broth was split into six parts, which served as the inoculum for biologically independent replicates to determine maximum caproic acid concentration (*n* = 3) and maximum rate of caproic acid production (*n* = 3).

#### Maximum rate of caproic acid production test

This test was performed (*n* = 3) in 50-mL Falcon tubes, each containing 30 mL of elongation broth. Half of the solution was replaced with effluent from the fermentation test every day for 4 days, for each replacement it was expressed as 1 cycle. From the 5th to 8th cycle, cells were retained by centrifugation. Before replacement of solution, the Falcon tubes were centrifuged at 5000*g* for 5 min to retain the cells in the elongation broth. The tubes were then left unattended for 1 week. After that, the operation resumed with cell retention for the 9th to 14th cycle. Samples were collected at 0, 1 and 24 h for carboxylic acids analysis. 1 mL of sample was collected for volatile suspended solid analysis, before the solution was centrifuged and replaced.

#### Maximum concentration of caproic acid test

The test was performed (*n* = 3) in 50-mL Falcon tubes, each containing 30 mL of elongation broth. 1.135 mL of 50% sodium lactate (VWR) solution was added to the elongation broth to give an extra 20 g L^−1^ lactate in the tubes. In addition to the lactate coming from the lactic acid fermentation, the resulting lactate concentration in the tubes was approximately 25 g L^−1^. Samples were taken at 0, 2, 4, 6, 8, 12, 24 and 48 h for carboxylic acids analysis.

### Electrochemical extraction

The electrochemical cell was constructed from Perspex™, consisting of an anode chamber (20 × 5 × 0.3 cm^3^) and a cathode chamber (20 × 5 × 2.6 cm^3^), separated by an anion exchange membrane (Fujifilm Manufacturing B.V., Netherlands) with a surface area of 0.01 m^2^. The cathode was an AISI Type 316L stainless steel felt (20 × 5 × 0.15 cm^3^) with 1-mm wire thickness (LierFilter Ltd., China), and the anode was a mixed metal oxide iridium oxide-coated titanium electrode (IrO_2_/TaO_2_: 0.65/0.35), 20 cm × 5 cm, with a centrally attached, perpendicular current collector (Magneto Special Anodes BV, The Netherlands). The cathode chamber was filled with effluent from the elongation system, and the anode chamber was filled with tap water. The extraction was performed in batch, a recirculation flow rate of 1.67 mL s^−1^ was maintained for both cathode and anode chambers to ensure mixing. 0.4 A of current was applied using a potentiostat (VSP, Biologic, France) in chronopotentiometry mode to drive the extraction operation, resulting in a current density of 25 A m^−2^ (membrane area). The pH of cathode chamber was controlled at pH 5.5 ± 0.3 by electrochemical water reduction and dosing of 2 M sulphuric acid solution with a pH controller.

### Kolbe electrolysis

The Kolbe electrolysis process was performed according to the study of Wadhawan et al. [35]. 50-mL one chamber dimerization reactor consisted of a spiral wire platinum anode (0.5 mm diameter, 3 cm^2^ surface area, Bio-Logic, France), a stainless steel plate cathode (12.5 cm^2^ surface area, height 5 cm × length 2.5 cm, and width 0.5 cm), and an Ag/AgCl reference electrode (filled with 3 M KCl solution). The chamber was filled with either control (0.5 M sodium sulphate in distilled water), synthetic solution (0.1 M caproic acid and 0.5 M sodium sulphate) or real solution (electrochemical extraction anolyte, containing 26 mM caproic acid), with pH adjusted to 7. Before electrolysis, cyclic voltammetry (CV, 10 mV s^−1^), with ohmic drop compensation, was performed on the broth using a potentiostat (SP-50, BioLogic, France), at 25 °C. Kolbe electrolysis experiments were performed at chronopotentiometry mode at a fixed current density of 0.133 A cm^−2^ (*i* = 0.4 A) for 24 h. Liquid phase of reactor was sampled at time 0, 2, and 24 h for analysis.

### Bacterial community analysis

#### DNA extraction

DNA extraction was performed as previously reported [5]. Samples were taken from initial inoculum, initial substrate and end of fermentation broth for analysis. 1.5 mL samples were centrifuged at 11,000*g* for 300 s in a 2-mL Lysing Matrix E tube (Qbiogene, Alexis Biochemicals, Carlsbad, CA). Pelleted cells were re-suspended in 1 mL of lysis buffer containing Tris/HCl (100 mM at pH 8.0), 100 mM EDTA, 100 mM NaCl, 1% (w vol^−1^) polyvinylpyrrolidone and 2% (w vol^−1^) sodium dodecyl sulphate. Cells were lysed using 0.2 cm^3^ beads of 0.1 mm size in a Fast Prep-96 homogenizer for 40 s at 1600 rpm twice. Samples were centrifuged at 18,000*g* for 60 s at room temperature and washed with phenol/chloroform (1:1) and chloroform. After centrifugation, nucleic acids (supernatant) were precipitated with 1 volume of isopropanol at −20 °C and 1:10 volume of 3 M sodium acetate. After centrifugation and washing with 80% ethanol, the pellet was re-suspended in 20 µL of Milli-Q water. The quality and quantity of DNA samples were analysed using Illumina sequencing primers by polymerase chain reaction (PCR). Amplified sequences were separated by electrophoresis on 1% agarose gels.

#### DNA sequencing and bioinformatics processing

DNA sequencing was performed as previously reported [5]. The V3–4 region of the bacterial 16S rRNA gene was sequenced with Illumina sequencing Miseq v3 Reagent kit (http://www.illumina.com/products/miseq-reagent-kit-v3.ilmn, by LGC Genomics GmbH, Berlin, Germany) using 2 × 300 bp paired-end reads, and primers 341F (5′-NNNNNNNNTCCTACGGGNGGCWGCAG) and 785R (5′-NNNNNNNNTGACTACHVGGGTATCTAAKCC). Each PCR included DNA extract (~5 ng), forward and reverse primer (~15 pmol for each) and MyTaq buffer (20 μL containing 1.5 units MyTaq DNA polymerase (Bioline) and 2 μL of BioStabII PCR Enhancer). 8-nt barcode sequence was performed for both forward and revers primers of each sample. PCRs were carried out for 96 °C pre-denaturation for 120 s and 30 cycles using the following parameters: 96 °C for 15 s, 50 °C for 30 s and 72 °C for 60 s. DNA concentration of amplicons of interest was determined by gel electrophoresis. Amplicon DNA of each sample (~20 ng) were pooled for up to 48 samples carrying different barcodes. PCRs showing low yields were further amplified for 5 cycles. The amplicon pools were purified with one volume AMPure XP beads (Agencourt) to remove primer dimer and other small mispriming products, followed by an additional purification on MinElute columns (Qiagen). Each purified amplicon pool DNA (~100 ng) was used to construct Illumina libraries using the Ovation Rapid DR Multiplex System 1–96 (NuGEN). Illumina libraries were pooled and size selected by preparative gel electrophoresis. Sequencing was done on an Illumina MiSeq using v3 Chemistry (Illumina).

16S rRNA sequence analysis was performed with mothur community pipeline and clustering into operational taxonomic units (OTUs). The analysis was initiated by clipping 16S rRNA sequences from primers. The fragments after removing the primer sequences were combined into forward and reverse primer orientation sequences. The sequences were then processed to remove wrong size sequence and identify unique sequences. Sequences which were not matched or overhung were removed by aligning with a V3–V4 customized SILVA database v123. Chimera were removed using UCHIME algorithm. Taxonomical classification of sequences and removal of non-bacterial sequences were done using Silva database v123. OTU were picked by clustering at 97% identity level using the cluster split method.

### Analytical methods

Determination of fermentation products including organic acids (lactic acid, acetic acid, propionic acid, butyric acid) was performed with Metrohm ion chromatography equipped with Metrosep organic acids column and Metrosep organic acids guard column, and an ion chromatography conductivity detector, using 1 mM H_2_SO_4_ as eluent at flow rate of 0.0083 mL s^−1^, oven temperature at 35 °C, and 500 mM LiCl as regenerant for suppressor. Caproic acid in aqueous phase was measured by gas chromatography (GC-2014, Shimadzu^®^, The Netherlands) with DB-FFAP 123-3232 column (30 m × 0.32 mm × 0.25 µm; Agilent, Belgium) and a flame ionization detector. Total solids (TS), volatile solids (VS) and volatile suspended solids (VSS) were measured via the standard method [41]. The particle size of blended grass was determined using a Zeiss Axioskop microscope and Image-Pro Insight software. Phase-separated caproic acid and decane were measured using nuclear magnetic resonance (NMR) spectroscopy. ^1^H NMR and ^13^C NMR were performed at 400 and 100 MHz, respectively, on a Bruker Avance III Nanobay 400 MHz spectrometer. 400 µL DMSO-d6 was added to 25 mg of caproic acid and decane sample, stirred and transferred to an NMR tube. Quantification was performed relative to caproic acid and decane as standard (0.2 M in DMSO-d6), contained in a NORELL 100 µL capillary insert. ^1^H NMR experiments were run with 8 scans and 1-s relaxation delay.

## Additional file

**Additional file 1: Figure S1.** Bacterial community for (a) inoculum for elongation system, (b) maximum rate test, (c) maximum concentration test. **Figure S2.** Carboxylate profile of maximum caproic acid concentration test – elongation under excess lactic acid condition (error bars represent the standard deviation of triplicates, and they are consistently less than 6% (n = 3) and obstructed by the medallions). **Figure S3.** Phase separation of caproic acid and aqueous elongation broth.
