# Supplementary material for: Electricity-assisted production of caproic acid from grass
Source: Biotechnol Biofuels. 2017 Jul 11;10:180. doi: 10.1186/s13068-017-0863-4 (PMC5504738; doi:10.1186/s13068-017-0863-4)
Supplement: Supplementary file 1 — Additional file 1: Figure S1. Bacterial community for (a) inoculum for elongation system, (b) maximum rate test, (c) maximum concentration test. Figure S2. Carboxylate profile of maximum caproic acid concentration test – elongation under excess lactic acid condition (error bars represent the standard deviation of triplicates, and they are consistently less than 6% (n = 3) and obstructed by the medallions). Figure S3. Phase separation of caproic acid and aqueous elongation broth. [file 13068_2017_863_MOESM1_ESM.docx]

Production of caproic acid and decane from grass

Way Cern Khor, Stephen Andersen, Han Vervaeren, Korneel Rabaey^‡^

Ghent University, Department of Biochemical and Microbial Technology, Centre for Microbial Ecology and Technology (CMET), Coupure Links 653, 9000 Gent, Belgium

^‡^**Corresponding author**: Korneel Rabaey

Email: Korneel.Rabaey@UGent.be

Tel: +32 09 264 59 76

Fax: +32 09 264 62 48


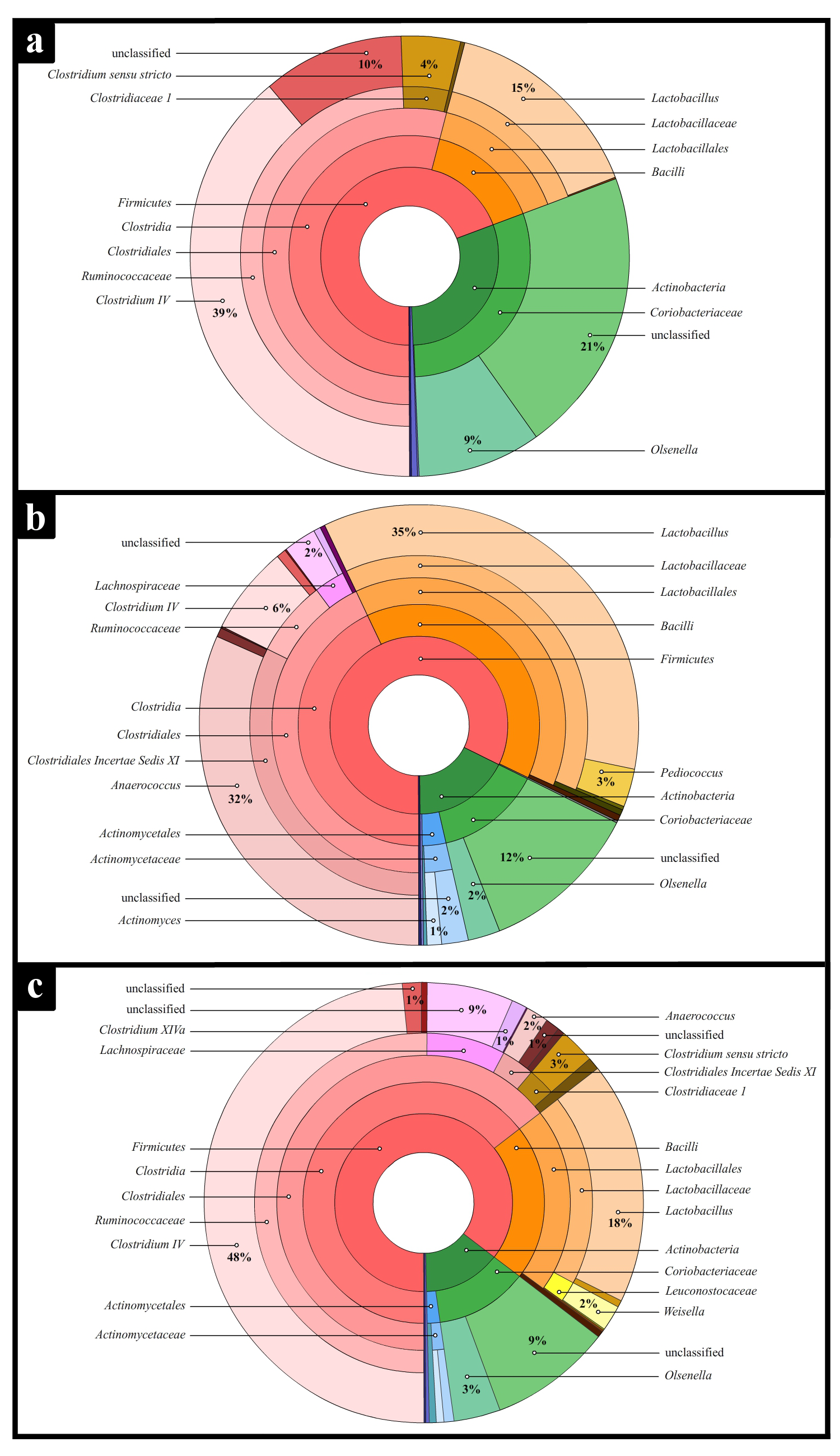


Fig. S1 Bacterial community for (a) inoculum for elongation system, (b) maximum rate test, (c) maximum concentration test


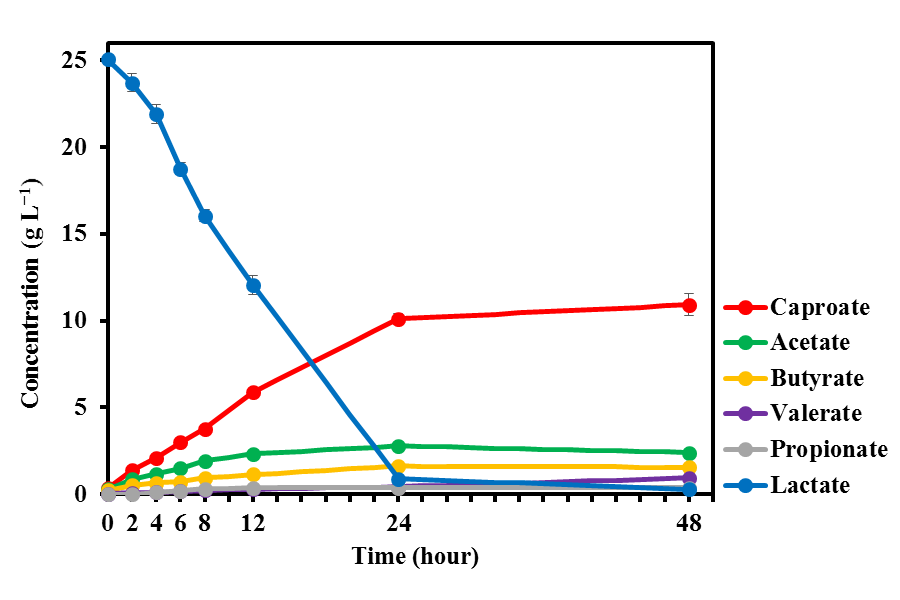


Fig. S2 Carboxylate profile of maximum caproic acid concentration test – elongation under excess lactic acid condition (error bars represent the standard deviation of triplicates, and they are consistently less than 6% (n = 3) and obstructed by the medallions)


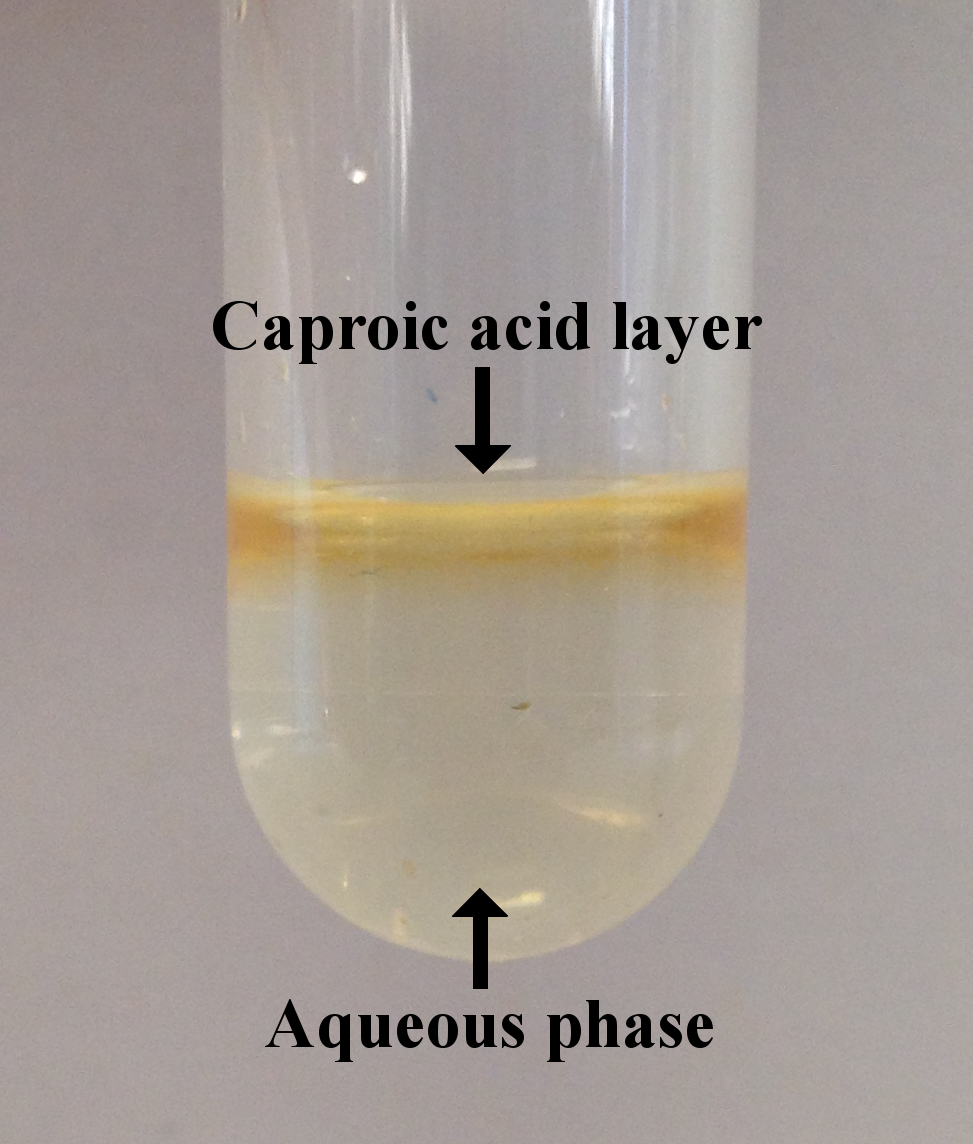


Fig. S3 Phase separation of caproic acid and aqueous elongation broth
